# Supplementary material for: 10-year level, trends and socio-demographic disparities of obesity among Ghanaian adults—A systematic review and meta-analysis of observational studies
Source: PLOS Glob Public Health. 2024 Jan 25;4(1):e0002844. doi: 10.1371/journal.pgph.0002844 (PMC10810441; doi:10.1371/journal.pgph.0002844)
Supplement: Attachment — Submitted filename: Response to Reviewers.docx [file pgph.0002844.s003.docx]

21^ST^ DECEMBER, 2023.

THE EDITOR,

PLOS Global Public Health

**REBUTTAL LETTER ON COMMENTS FROM REVIEWERS AND EDITOR**

I wish to begin by thanking the Reviewers and Editor for their efforts and time spent on reviewing our manuscript and especially providing us with very useful feedback.

We would like to admit that most of the comments are genuine and we concede to the lapses identified in the work.

Most of the suggestions and comments have been looked at, leading to some revisions in the manuscript which we would like to provide below as my responses.

**Review comments and actions taken**

| **Review comments** | **Action taken/Response** |
| --- | --- |
| Editor Comments |  |
| 1) it is imperative for the validity of your systematic review and meta-analysis that you clearly articulate compliance with the COSMOS-E and PRISMA guidelines. Please include a detailed statement in the methodology section confirming adherence to these guidelines and ensure that all the relevant criteria are met | Action has been taken on this as shown in Lines 111 - 114 |
| 2) Your utilization of the AXIS tool for assessing quality and risk of bias is noted. However, the manuscript would benefit from a deeper explanation of how these assessments influenced your analysis. For instance, consider discussing the incorporation of quality scores within a quality effects model, as proposed by Doi and Thalib (2008) | Quality scores have been incorporated in the quality assessment of the studies resulting in the calculation of quality indices (Qi) for each study.  A quality effects model has now been used to determine the pooled estimates for all the study outcomes |
| The process of managing abstracts and eliminating duplicates is crucial in systematic reviews. Could you please elucidate the tools (such as Rayyan) or methods employed to this end? Moreover, details regarding the independent screening process and how discrepancies were resolved would greatly contribute to the transparency of your study's methodology. | The process of managing abstracts and eliminating duplicates was facilitated by the use of the Covidence software as started in Line 129. No additional tools were used. Discrepancies were resolved by consensus as stated in line 177 and 178 |
| Assessment of Heterogeneity and Publication Bias: You've identified a high level of heterogeneity and publication bias within the included studies. To address this, we suggest exploring the use of the Doi plot and the Luis Furuya-Kanamori (LFK) index for a more robust visualization and quantification of asymmetry in study effects. Additionally, forest plots would be valuable for graphically presenting the meta-analysis results, including information on heterogeneity levels | Publication bias has now be assessed using Doi plot and LFK index as indicated in Lines 209 and 210 |
| Reviewer #1 |  |
| 1) Congratulations to the study for a great job done with this systematic review on the prevalence of obesity and overweight in Ghana. |  |
| 2) Since the search duration was updated in January 2023, please correct the search period to Oct 2022 to Jan 2023. | Suggested correction done as reflected in Line 23 and Line 120 |
| 3) Line 129: why were other studies included irrespective of the metabolic status of the participants? What value addition or relevant information were gleaned from these other studies? If line 135 is making reference to the studies referred to on line 129, then please rephrase to make the statement clearer that relevant information in these studies would be used to compute prevalence. | The purpose of the statement in Line 129 was to address the inclusion criteria of studies with respect to the type of study participants under the PICOS criteria. We did not exclude any studies that reported the prevalence of overweight/obesity because the study participants had any metabolic disorder eg. diabetes. We rather included all and performed a sub-group analysis of prevalence among diabetes patients and non-diabetes patients.  The statement in line 129 has been rephrased for purposes of clarity |
| 4) Lines 211 and 212: can pass as a limitation. There should be a period at the end of line 212. | Line 212 has been taken off and discussed as part of the study limitations. This is reflected in Lines 474 - 476 |
| 5) Line 256: Table 2 didn’t show any level of significance for the variable “marital status” | There was an omission. The level of significance for the “marital status” variable has now been added. |
| 6) Figure 11: What could have accounted for the change in trends for the two variables in 2017/18 especially being overweight? | This will need further exploration. There are no known explanations yet |
| REVIEWER # 2 - METHODS |  |
| 1. Please indicate whether the authors followed the PRISMA guideline. | This has been don |
| REVIEWER # 2 - ANALYSIS |  |
| 1. The Statistical values of publication bias analysis need to be in the Funnel plot figures or result tables. | Statistical values have been incorporated into Funnel plot figures |
| 2. Please add a Table for the result of Proportion subgroup analysis (Variation in Obesity Proportion according to subgroups) | This data/results is shown in Figure 12 |
| 3. Please add a Figure for "overweight and obesity diabetes patients in Ghana." | Figure has been added |
| 4. Figure 12 is empty. Please be sure to see Figure 12 again. | Correction has been done. Figure 12 is now fully visible |
| REVIEWER # 3 - ABSTRACT |  |
| Line 35: needs corrections on the 25.6kb-2 by adding “m” so that it will be 25.6kgm-2 | Correction has been done |
| REVIEWER #3 - INTRODUCTION |  |
| Line 51: since in Line 53 the authors have used SSA to mean Sub-Saharan Africa; they need to insert the abbreviation SSA in brackets in line 51 just after Africa to be as follows: Sub-Saharan Africa (SSA) has seen more…... | Abbreviation has been inserted in brackets as required in line 51 |
| Line 67: I suggest to the authors to replace “low- and middle- income countries (LMICs) with its abbreviation LMICs since it was already abbreviated in Line 50. | Correction has been done |
| Line 93: (Meanwhile, the prevalence of overweight and obesity continuous to rise in Ghana over the), I hope the authors meant “continues” instead of “continuous”. | Correction has been done |
| REVIEWER #3 - METHODS |  |
| Line 110: I suggest to the authors to edit “PUBMED” to be “PubMed”. | Correction has been done |
| REVIEWER #3 - RESULTS |  |
| Line 354: I suggest to the authors to replace “cardiovascular diseases” with its abbreviation “CVDs”. | Correction has been done |
| REVIEWER #3 - DISCUSSION |  |
| Lines 377–378: I suggest to the authors to replace “sub-Saharan Africa” with its abbreviation SSA used in Lines 51 & 53 | Correction has been done |
| Line 384: (years (2019/2020) confirming that overweight and obesity continuous to rise in Ghana as the), I hope the authors meant “continues” instead of “continuous”. | Correction has been done |
| Line 429: (levels based on gender and urbanization suggests that Ghana continuous to be in Stage 1 of the), I hope the authors meant “continues” instead of “continuous”. | Correction has been done |
| Line 437: I suggest to the authors to replace “Sub Saharan Africa” with its abbreviation SSA. | Correction has been done |
| Line 441: I suggest to the authors to replace “non-communicable diseases” with its abbreviation NCDs which was already abbreviated in Line 54. | Correction has been done |
| Line 458: (poorer than the rest of rest of the country with the wealthier regions being the southern regions), The authors need to DELETE the repeating words “rest of”. | Repeating words have been deleted |
| REVIEWER #3 – FIGURE 12 |  |
| The figure seems to be incomplete in terms of displaying the information on disparities. Therefore, I suggest to the authors to recheck it; & | Figure 12 (now figure 8) now displays all the information |
| I also suggest to the authors to ensure that it (Fig.12) is linked in the text to enable a reader to follow through easily. | A linkage to figure 12 (now figure 8) has now been done in the text in Lines 307 – 308. |

Thank you.

MUSTAPHA TITI YUSSIF

CORRESPONDING AUTHOR
